# The Relation of Homœopathy to Hahnemann

**Published:** 1884-05

**Authors:** Ad. Lippe

**Affiliations:** Philadelphia, Pa.


### THE RELATION OF HOMŒOPATHY TO HAHNEMANN.

AD. LIPPE, M. D., PHILADELPHIA.

*The British Journal of Homœopathy* opens the present year with a leading paper on "The Relation of Homœopathy to Hahnemann," by Dr. Hughes. As it comes to us as an introductory lecture to a course on the principles of Homœopathy delivered in the London Homœopathic Hospital Medical School, and as it is misleading, it becomes an unpleasant duty to expose the erroneous arguments offered, which, if accepted, would pervert the true homœopathic healing art into eclecticism.

The great object of the learned lecturer is to show that the formula, *similia similia curantur*, adopted by Hahnemann, is not the correct homœopathic formula, but should read *similia similibus curentur*. His argument is that *curantur* is unwarrantable if implying that all cure is wrought under the law of the similars, and to fortify his position he erroneously claims that Homœopathy is a *method*, not a *doctrine* or a *system*, and that Homœopathy does not deny that there are other aspects of disease and drug-action than the formula *similia similibus curantur* implies, nay, necessitates.

Either the law of the similars is correctly expressed by the adopted formula, *similia similibus curantur*, or else the law of the similars is not a law at all, only a method among other methods of cure, belonging exclusively to eclecticism, and then very properly and for that purpose exclusively expressed in good Latin by the "*similia similibus curentur*." Either Homœopathy is applicable to the cure of all and every non-surgical disease, or it is not applicable in all such cases. Hahnemann says *it is* and Hughes contends that *it is not*. Hahnemann, in the *Organon* of the healing art, clearly defines the position he takes in paragraph 54: "I have before remarked (pages 43 to 49) that the course pursued by Homœopathy is the only correct one. Because of the three ways to apply medicines in diseases it is the only direct one leading to a mild, certain, and durable cure, without either injuring the patient or diminishing his strength. The pure homœopathic mode of cure is the only correct, the only direct, and the only possible means to be employed by human skill, as surely as it is possible to draw but one straight line between two given points." Here is a flat denial by Hahnemann that there is or can be any other mode of cure than the homœopathic. The illustration Hahnemann uses is simple, comprehensible, and universally accepted. Between two given points but *one* straight line can be drawn. As true as this fundamental mathematical law is, just as true is it that the only way all diseases are cured is under the guidance of the law of the similars. And this law is, as our good friend, Dr. P. P. Wells, so forcibly expresses himself, as wholly mandatory. It commands, and a natural "law can therefore never be degraded to a mere rule of practice."

In order to mislead, one of the boldest attempts to misrepresent Hahnemann is made when Dr. Hughes asserts that Hahnemann proved Cinchona officinalis to find out whether, like other febrifuges, it was febrigenic at all; and that his result was to find it productive of all symptoms, both general and characteristic, of the intermittent paroxysm. Where did Hahnemann say that? The *Materia Medica Pura* (*not* a pathological picture-book) has seen a new translation by R. E. Dudgeon, M. D., with annotations by Richard Hughes, L. R. C. P. E. If the learned annotator will compare what Hahnemann says in his admirable preface to China officinalis he must retract his misleading assertion just quoted. Hahnemann says :*

"As long ago as the year 1790 (See W. Cullen's *Materia Medica*, Leipzig, bei Schwickert i. i. p. 109 note). I made the first pure trial with Cinchona

---

* Page 408, in Dr. Dudgeon's translation of the *Mat. Med. Pura.*

bark upon myself, in reference to its power of exciting intermittent fever. Cullen had asked the very pertinent question, 'Under what circumstances does China cure intermittent fever.'"

When Hahnemann made the first pure trial with Cinchona bark upon himself in reference to its power of exciting intermittent fever it was undoubtedly his purpose to ascertain the fact that this bark could excite on the healthy organism intermittent fever symptoms; he had no doubt then a conviction that such must be the case, or else that bark could not possess at times the power to cure intermittent fever; *then* came the question, *Under what circumstances* can, must, it cure? And if our learned friend will read on in that above-quoted foot-note, he will, maybe, see daylight. Hahnemann says:

"With this first trial broke upon me the dawn that has since brightened into the most brilliant day of the medical art; that it is only in virtue of their power to make the healthy human being ill that medicines can cure morbid states, and, indeed, only such morbid states as are composed of symptoms as the drug to be selected for them can itself produce in similarity on the healthy. This is a truth so incontrovertible, so absolutely without exception, that all venom poured out on it by the members of the medical guild, blinded by their thousand-years' old prejudice, is powerless to extinguish it, etc."

What Hahnemann says further on in this admirable, clearly written preface to China is as true to-day as it was then. Even the characteristic indications for the use of China are unsurpassed by anything published since. He who will take the time to attentively read these indications will not fail to cure the few cases curable by China. He will, in the course of time, find, for instance, a morbid state excited intensely, aggravated by merely touching the part, or slightly moving it, and as China is really the only known medicine causing a similar symptom in its primary action, a single appropriate dose will very speedily cure that case.

The ardent desire of Richard Hughes & Co. to build up a new system based on "Progressive Pathology" and painted up with seducive colors under the banner *similia similia curentur*, is expressive of the true inwardness of these illogical men.

We have at present three sets of men before the world, all professing to be homœopaths, all three quoting Hahnemann as their "authority." There are the Old Guard and the readers of Hahnemann's works who honestly and diligently, understandingly and painstaking, have followed him, and who honestly proclaim that their success in combating diseases has increased as they more strictly adopted Hahnemann's methods under the formula, *similia similibus curantur*. Likes *are* cured by likes.

There are the misleaders who fable of a variety of methods, despise any "law," and who *never* (although frequently asked),* have illustrated their untenable position, their formula is *similia similibus curentur*. Likes *may* be cured by likes. Hahnemann was a ripe classical scholar, and when he wrote *curantur* (are) he wrote down "a law." Whoever says *curentur* (may be) omits to say when, and under what circumstances the law of the similars (Homœopathy) is applicable and what next? What other methods can be applied? And under what circumstances? All is sadly mixed up. Why not be honest and say we are wiser than was the Master, and we are eclectics, and *we* will give the world something to laugh at—a pathological picture-book? For the information of Richard Hughes & Co., and their boasted huge majority, be it known, that we are far ahead in the United States, and that in the reprint of the *Encyclopædia Britannica* in the United States these gentlemen will find a very strong paper on Homœopathy, written by a "scholar," who treats the subject becomingly.

There are others calling themselves also homœopaths who claim that Hahnemann advocated the formula, *æqualia æqualibus curantur*. Lux tried that folly and failed. So will it be with the present imitators of so glaring an absurdity.

The sum and substance of our paper is to show that Hahnemann was the founder of Homœopathy with his own formula, *similia similibus curantur;* and that Richard Hughes, with his new formula, *similia similibus curentur*, has departed from Homœopathy hopelessly. *Bon voyage.*

*State a case of sickness in which the law of *similia similibus curantur* was honestly applied, and after failing to cure it, was finally *cured* by some other method of cure.

